# Reviewed article: Oliveira VO, Sampaio ALN, Júnior RWM. Temporal trend of vasectomies in Brazil and its regions by age group and race/skin color: a temporal analysis from 2013 to 2022. Epidemiol Serv Saude. 2025:34;e20240209

**DOI:** 10.1590/S2237-96222024v34e20240209.b

**Published:** 2025-05-12

**Authors:** Divanise Suruagy Correia

**Table:** 

Rating	Excellent	Good	Average	Below average	Poor
Interest	✓				
Quality	✓				
Originality		✓			
Overall	✓				

**Table:** 

Questionnaire	Yes	No	Not applicable
Does the manuscript contain new and significant information to justify publication?	✓		
Does the Abstract (Summary) clearly and accurately describe the content of the article?	✓		
Is the problem significant and concisely stated?	✓		
Are the methods described comprehensively?	✓		
Are the interpretations and conclusions justified by the results?	✓		
Is adequate reference made to other work in the field?	✓		

## Manuscript Structure:

Length of article is: Adequate

Number of tables is: Adequate

Number of figures is: Adequate

Please state any conflict(s) of interest that you have in relation to the review of this paper (state “none” if this is not applicable): None

## Comments

Excelente trabalho, sugiro retirar apenas o objetivo da conclusão.

